# New Wasps of Maimetshidae (Hymenoptera: Ceraphronoidea) from the Mid-Cretaceous Myanmar Amber

**DOI:** 10.3390/insects16030237

**Published:** 2025-02-23

**Authors:** Longfeng Li, Dmitry S. Kopylov, Alexandr P. Rasnitsyn, Jingtao Yang, Chungkun Shih, Dong Ren

**Affiliations:** 1 Institute of Vertebrate Paleontology, College of Life Science and Technology, Gansu Agricultural University, Lanzhou 730070, China; yangjt@gsau.edu.cn; 2 Institute of Zoology, Almaty 050060, Kazakhstan; aeschna@yandex.ru; 3 Paleontological Institute, Russian Academy of Sciences, Profsoyuznaya Street, 123, Moscow 117647, Russia; alex.rasnitsyn@gmail.com; 4 Natural History Museum, London SW7 5BD, UK; 5 College of Life Sciences, Capital Normal University, 105 Xisanhuanbeilu, Haidian District, Beijing 100048, China; chungkun.shih@gmail.com (C.S.); rendong@cnu.edu.cn (D.R.); 6 Department of Paleobiology, National Museum of Natural History, Smithsonian Institution, Washington, DC 20013, USA

**Keywords:** Apocrita, new genera, new species, taxonomy, Burmese, Kachin

## Abstract

One new genus and five new species of the extinct family Maimetshidae from mid-Cretaceous Kachin amber are described based on five well-preserved specimens as follows: *Maimetshasia nova* Li, Kopylov and Rasnitsyn, sp. nov.; *Maimetshasia engeli* Li, Kopylov and Rasnitsyn, sp. nov.; *Guyotemaimetsha perrichoti* Li, Kopylov and Rasnitsyn, sp. nov.; *Guyotemaimetsha ortegablancoi* Li, Kopylov and Rasnitsyn, sp. nov.; and *Crucimaimetsha nigra* Li, Kopylov and Rasnitsyn, sp. nov. A new generic synonymy is proposed: *Turgonalus* Rasnitsyn, 1990 (=*Turgonaliscus* Engel, 2016, syn. nov. and = *Ahiromaimetsha* Perrichot, Azar, Nel and Engel, 2011, syn. nov.); *Guyotemaimetsha* Perrichot, Nel and Néraudeau, 2004 (=*Burmaimetsha* Perrichot, 2013, syn. nov.); *Afrapia*, Rasnitsyn and Brothers, 2009 (=*Afromaimetsha*, Rasnitsyn and Brothers, 2009, syn. nov.); and *Iberomaimetsha nihtmara* Ortega-Blanco, Delclòs and Engel, 2011 are transferred to *Afrapia,* Rasnitsyn and Brothers, 2009. This results in the following new combinations: *Turgonalus cooperi*, Rasnitsyn and Jarzembowski, 1998, comb. resurr.; *Turgonalus najlae* (Perrichot, Azar, Nel & Engel, 2011), comb. nov; *Afrapia robusta* (Rasnitsyn & Brothers, 2009), comb. nov; and *Afrapia nihtmara* (Ortega-Blanco, Delclòs & Engel, 2011), comb. nov. The occurrence of various genera of Maimetshidae in amber and compression fossil and the morphological differences of the forewings in all the fossil species are provided, which highlights a high level of genus-level diversity among Mesozoic maimetshids. The key to genera was updated.

## Simple Summary

New fossil taxa of the extinct family Maimetshidae are described and figured from mid-Cretaceous Kachin amber. Forewing venations and localities and horizons of various genera of Maimetshidae in amber and compression fossils are summarized for comparison, suggesting that Maimetshidae have a high degree of venational diversity.

## 1. Introduction

Maimetshidae, an extinct family of Apocrita within Hymenoptera, was first established based on a single genus and species, *Maimetsha arctica*, described from the Upper Cretaceous Taimyr amber [1]. Half a century later, the family has expanded to include 23 species across 12 genera from the Cretaceous, with a wide geographical distribution. Records now span Russia [2,3,4], England [5], France [6,7], Botswana [8], Lebanon [7,9], Spain [7], Canada [7], and Myanmar [10]. Notably, maimetshids have been recorded in all major Cretaceous amber deposits except those of New Jersey (Table 1). Conversely, they remain poorly represented in Cretaceous insect-rich rock deposits, with only a few findings from England [5], Siberia [3], and Botswana [8]. This scarcity likely reflects a broader trend where small insects are less frequently preserved or detected as rock fossils.

The generic diversity of Maimetshidae has necessitated a more detailed taxonomic organization. Engel [9] proposed a division into two subfamilies, Maimetshinae and Zorophratinae, and further divided Maimetshinae into tribes Maimetshini and Ahiromaimetshini, covering 15 genera, 13 of which were monotypic. While we accept Engel’s subfamilies, recent discoveries in Kachin amber suggest the need to revisit the current system due to the high prevalence of monotypic genera. We propose some new synonymies, which, among other changes, result in Maimetshini and Ahiromaimetshini becoming monotypic. These tribes are based on a single diagnostic character each (Maimetsha lacks 2r-m whilst 3r-m is preserved, and Ahiromaimetsha lacks 2-Rs+M), which seems insufficient to justify tribal distinctions under current criteria.

Cretaceous amber from northern Myanmar has yielded a remarkable diversity of hymenopteran wasps, with over 350 species in 202 genera across 70 families reported over the past century [11]. These include both extinct and extant taxa, such as Evaniidae and Aulacidae [12], Gasteruptiidae [13], Pelecinidae [14,15], Ichneumonidae [16], Formicidae [17], and Stephanidae [18]. The first fossil Maimetshidae from Myanmar included two genera and two species [10]. In this study, we describe five new species across three genera (including one new genus) from the mid-Cretaceous Myanmar amber.

## 2. Material and Methods

**Figure 1:**
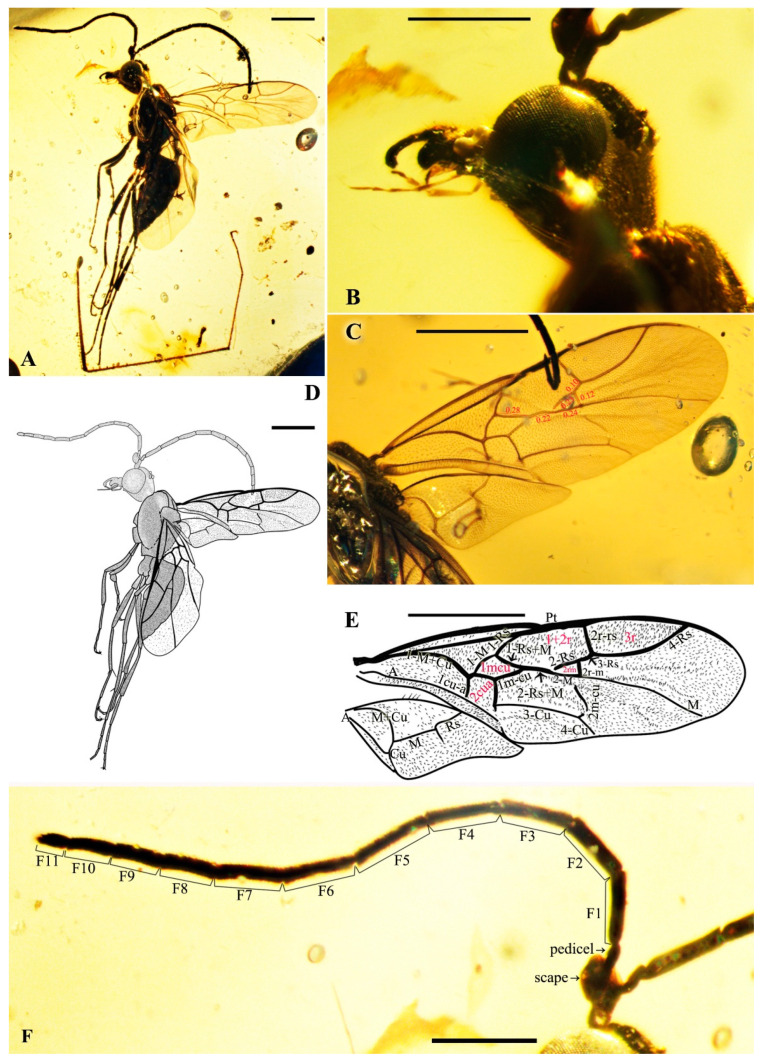
*Maimetshasia nova* Li, Kopylov and Rasnitsyn, sp. nov. Holotype GAU-HYM-MA-2016009. (**A**,**D**) Habitus, lateral views. (**B**) Head. (**C**,**E**) Wings. (**F**) Antennae. Scale bars = 1 mm (**A**,**C**–**E**); =0.5 mm (**B**,**F**).

The amber samples analyzed in this study originate from the Hukawng Valley, Kachin State, northern Myanmar, located approximately 100 km southwest of the village of Tanai ([19], Figure 1). Radiometric dating indicates an early Cenomanian age (98.79 ± 0.62 Ma) for the Kachin amber, based on zircons from volcanic clasts within the amber-bearing sediments [20]. Additionally, ammonites discovered in the amber-bearing bed and within the amber itself support a late Albian to early Cenomanian age for these deposits [21,22].

All type specimens reported herein are deposited at the College of Life Science and Technology, Gansu Agricultural University, Lanzhou City, Gansu Province, China. The five samples were examined and photographed using a Nikon SMZ 25 dissecting microscope and a Nikon DS-Ri2 digital camera system (Nikon Precision Shanghai Co., Ltd., GAU, Lanzhou in China). The morphological terminology follows Rasnitsyn and Brothers [8], with some modifications.

## 3. Results

Systematic palaeontology.

Order Hymenoptera Linnaeus, 1758.

Suborder Apocrita Gerstaecker, 1867.

Superfamily Ceraphronoidea Haliday, 1833.

Family Maimetshidae Rasnitsyn, 1975.

Subfamily Maimetshinae Rasnitsyn, 1975.

Genus *Turgonalus* Rasnitsyn, 1990.

Type species *Turgonalus minor* Rasnitsyn, 1990.

=*Turgonaliscus* Engel, 2016, syn. nov.

Type species *Turgonalus cooperi* Rasnitsyn and Jarzembowski, 1998.

=*Ahiromaimetsha* Perrichot, Azar, Nel and Engel, 2011, syn. nov.

Type species *Ahiromaimetsha najlae* Perrichot, Azar, Nel and Engel, 2011.

Diagnosis. 2-Rs+M absent or very short, 1m-cu effectively confluent with 2-Rs.

Species included. *Turgonalus minor* (type species), *Turgonalus cooperi*, and *Turgonalus najlae* comb. nov. [7].

Remarks. The synonymy above is due to the comparatively minor differences between the three species in question. For their distinction, refer to the key provided below:

Genus *Afrapia* Rasnitsyn and Brothers, 2009;

Type species *Afrapia globularis* Rasnitsyn and Brothers, 2009;

=*Afromaimetsha* Rasnitsyn and Brothers, 2009;

Type species *Afromaimetsha robusta* Rasnitsyn and Brothers, 2009.

Diagnosis. Both 1r-m and 2r-m present (2r-m tubular or nebulous, but always distinct); cell 2rm petiolate, with 2r-m longer than petiole; 2Rs+M subequal to, or distinctly longer than 1m-cu; 4-Rs as long as, or shorter than 3+4-M.

Species included. Afrapia globularis (type species), Afrapia variicornis, Afrapia robusta [8] comb. nov, and Afrapia nihtmara [7], comb. nov.

Remarks. The synonymy above is due to the similarity between *A. globularis*, *A. variicornis*, and *A. robusta*. *A. nihtmara* was described under *Iberomaimetsha* by Ortega-Blanco, Perrichot, and Engel (2011), albeit with considerable doubts due to its deviated wing venation as noted by Perrichot et al. [7]. In our opinion, it fits better within *Afrapia*, though not without some reservations. A particular issue arises with the paratype of *Iberomaimetsha nihtmara*, described by Ortega-Blanco et al. Based on the photographs provided in the description, its wing venation significantly differs from that of the holotype.

Genus *Maimetshasia* Perrichot, 2013.

Type species *Maimetshasia kachinensis* Perrichot, 2013.

Diagnosis. Forewing with 2-Rs+M shorter than 1-Rs+M, 1-M and 1m-cu distinctly diverging, cell 2rm petiolate, 2r-m longer than petiole (3-Rs), and 3r-m absent.

Species included. *Maimetshasia kachinensis* (type species), *Maimetshasia nova* Li, Kopylov and Rasnitsyn, sp. nov., and *Maimetshasia engeli* Li, Kopylov and Rasnitsyn, sp. nov.

*Maimetshasia nova* Li, Kopylov, and Rasnitsyn, sp. nov. (Figure 1).

urn:lsid:zoobank.org:act:C45DA7E4-84FB-4F13-B449-20898DAE50E4.

Type material. Holotype, Male, GAU-HYM-MA-2016009, well-preserved except the metasomal segments, which are not clearly distinguishable.

Etymology. The specific name is derived from the Latin word ‘nova’, meaning new, referring to the new record in Burmese amber.

Locality and horizon. Hukawng Village, Kachin State, Northern Myanmar; mid-Cretaceous.

Diagnosis. Antennae with 13 segments (vs. 16 segments in *M. kachinensis* and *M. engeli*). Forewing with 2r-m straight (vs. sinuous in *M. kachinensis*), forewing with 2-Rs+M, 2-Rs, and 2-M nearly equal in length (vs. 2-M longer than 2-Rs and 2-Rs longer than 2-Rs+M in *M. kachinensis* and *M. engeli*), and 2r-m almost coinciding with 2m-cu (distant from that in *M. kachinensis* and *M. engeli*); 1m-cu longer than 1-M (vs. shorter than 1-M in *M. kachinensis* and *M. engeli*).

Description. Male. Total body nearly dark except wings hyaline and hirsute. Body length about 4.0 mm long (from top of head to end of metasoma); forewing length, 3.3 mm and width, 1.0 mm; hind wing length, 1.5 mm and width, 0.7 mm. Head rounded, 0.9 mm long and 0.8 mm wide; compound eye almost circular, bulging, occupying 2/3 length of head, and 0.5 mm long and 0.5 mm wide; temples long; antenna filiform, about 3.2 mm long, with 13 segments, scape distinctly longer and wider than pedicel; mandibles extending, with few details visible, five maxillary palpomeres. Both mesosoma and metasoma laterally oval, longer than high. Legs slender, fore leg length (mm) with coxa, 0.38; trochanter, 0.24; femur, 0.79; tibia, 0.74; tarsus, 1.2. Midleg length (mm) with coxa, 0.38; trochanter, 0.38; femur, 1.0; tibia, 1.0; tarsus, 1.3. Hind leg length (mm) with coxa, 0.5; trochanter, 0.38; femur, 1.3; tibia, 1.19; tarsus, 1.79. Forewing with pterostigma very narrow, 1-Rs 1.05× as long as 1-M; 1cu-a antefurcal and shorter than 1-M; 2r-rs straight and long, more than 2× as long as 2r-m; 1-Rs+M 1.3× as long as 2-Rs+M, 2-Rs 2× as long as 3-Rs, 2r-m almost coinciding with 2m-cu, 2m-cu present.

**Figure 2:**
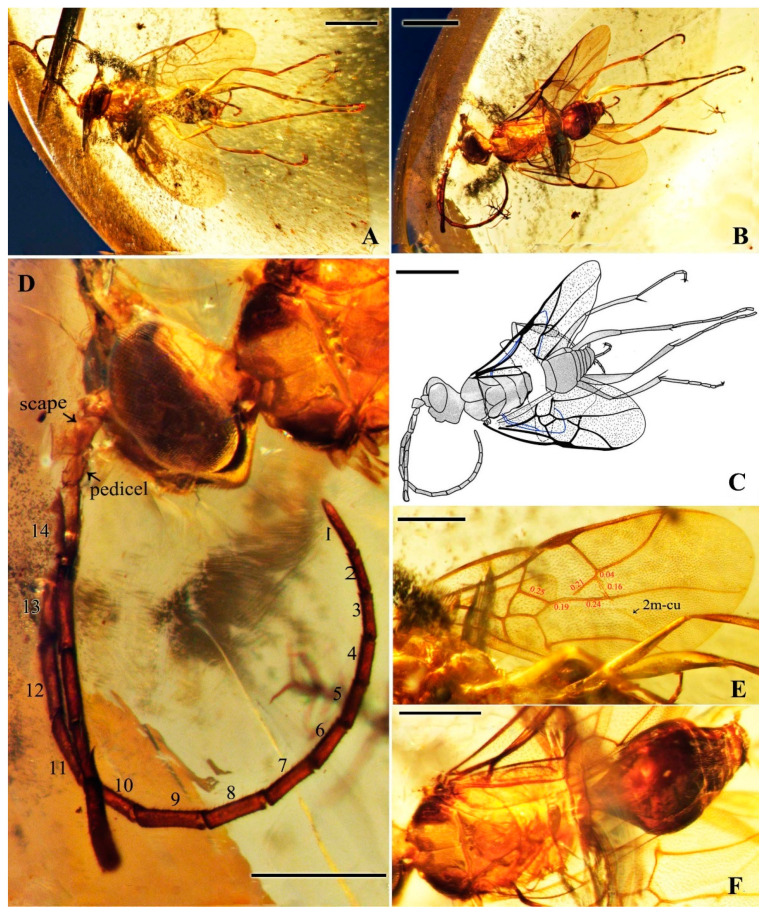
*Maimetshasia engeli* Li, Kopylov and Rasnitsyn, sp. nov. Holotype GAU-HYM-MA-2016013. (**A**–**C**) Habitus, dorsal and ventral (**B**) views. (**D**) Head. (**E**) Wings. (**F**) Mesosoma and metasoma. Scale bars = 1 mm (**A**–**C**); =0.5 mm (**D**–**F**).

*Maimetshasia engeli* Li, Kopylov and Rasnitsyn, sp. nov. (Figure 2).

urn:lsid:zoobank.org:act:C45DA7E4-84FB-4F13-B449-20898DAE50E4.

Type material. Holotype, Male, GAU-HYM-MA-2016013, well-preserved except partially obscured by debris from below and one antenna incomplete.

Etymology. The species is named in honor of the American paleoentomologist Michael S. Engel, who has made a significant contribution to the development of the taxonomy of Maimetshidae.

Locality and horizon. Hukawng Village, Kachin State, Northern Myanmar; mid-Cretaceous.

Diagnosis. Antennae with 16 segments (vs. 13 segments in *M. kachinensis*). Forewing with 1-Rs+M about 1.3× as long as 2-Rs+M (vs. more than 2× as long as 2-Rs+M in *M. kachinensis*), 3-Rs very short. Mandibles short (vs. long in *M. kachinensis* and *M. nova*).

Description. Male. Total body brown, wings hyaline and hirsute. Body length about 2.7 mm long (from the scape to the end of metasoma); forewing length, 2.6 mm; hind wing length, 1.1 mm. Head oval with 0.6 mm long and 0.8 mm high; compound eye large, egg-shaped, occupying 2/3 high of head; antenna with 16 segments, about 3.1 mm long, scape distinctly longer and wider than pedicel. Mesosoma slightly narrower than head in lateral view, pronotum long and divided by center line, mesonotum with notauli nearly as long as pronotum, metanotum slightly wider and longer than pronotum. Metasoma with seven visible segments, 2/3 base of first metasomal segment formed as wide petiole, and second metasomal segment distinctly longer and wider than remaining segments. Legs slender, fore leg length (mm): femur, 0.64; tibia, 0.58; tarsus, 0.74. Midleg length (mm): femur, 1.0; tibia, 0.8; tarsus, 1.1. Hind leg length (mm): femur, 0.9; tibia, 0.8; tarsus, 1.4. Forewing with pterostigma very narrow, 1-Rs nearly as long as 1-M; 2r-rs straight and long, more than 1.5× as long as 2r-m, 1-Rs+M longer than 2-Rs+M, 2-Rs more than 4× as long as long 3-Rs; 1m-cu present, 2m-cu nebulous. Hind wing only outline observed.

Genus *Guyotemaimetsha* Perrichot, Nel and Néraudeau, 2004.

Type species *Guyotemaimetsha enigmatica* Perrichot, Nel and Néraudeau, 2004.

=*Burmaimetsha* Perrichot, 2013.

Type species. *Burmaimetsha concava* Perrichot, 2013.

Diagnosis. Forewing with 2-Rs+M longer than 1-Rs+M, 1-M and 1m-cu not distinctly diverging, cell 2rm petiolate, 2r-m longer than petiole (3-Rs), 3r-m spectral or absent, at most visible as short stubs on, or just bends of, Rs and M at its place.

Species included. *Guyotemaimetsha enigmatica* (type species), *Guyotemaimetsha concave* comb. nov., *G. perrichoti* Li, Kopylov and Rasnitsyn, sp. nov., and *G. ortegablancoi* Li, Kopylov and Rasnitsyn, sp. nov.

Remarks. The above synonymy is because the new species described herein blur hiatus between the genera concerned.

**Figure 3:**
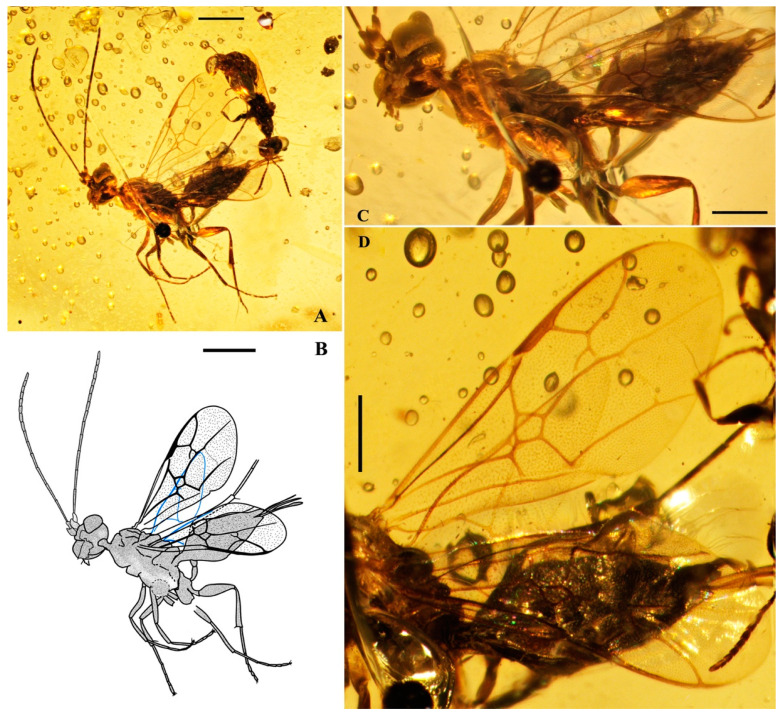
*Guyotemaimetsha perrichoti* Li, Kopylov and Rasnitsyn, sp. nov. Holotype GAU-HYM-MA-2016010. (**A**,**B**) Habitus, lateral views. Scale bars = 1 mm. (**C**) Head, mesosoma, and metasoma in lateral view. (**D**) Wings. Scale bars = 0.5 mm.

*Guyotemaimetsha perrichoti* Li, Kopylov and Rasnitsyn, sp. nov. (Figure 3).

urn:lsid:zoobank.org:act:40458C6B-9E75-4560-A31E-23AEE77D5D55.

Type material. Holotype, Female, GAU-HYM-MA-2016010, well-preserved except for some parts of mid- and hind coxae and the metasomal segments not clearly distinguished.

Etymology. The species is named in honor of the French paleoentomologist Vincent Perrichot, who has made an invaluable contribution to the study of the family Maimetshidae.

Locality and horizon. Hukawng Village, Kachin State, Northern Myanmar; mid-Cretaceous.

Diagnosis. Antennae with 16 segments. Forewing with 2-Rs and 3-Rs forming an obtuse angle, cell 1+2r longer than 3r, and cell 3rm as long as broad. First metasomal segment much narrower at base and ovipositor ca. 1× as long as hind tibia.

Description. Female. Total body dark except wings lighter. Body length about 3.86 mm long (from the base of antennae to the end of metasoma); forewing length, 2.83 mm; width, 1.24 mm. Hind wing length, 1.88 mm; width, 0.96 mm. Ovipositor sheath, 0.87 mm. Head transversely oval with 0.67 mm long and 0.83 mm wide; antenna filiform about 3.44 mm long, with 16 segments, scape distinctly longer and wider than pedicel. Mesosoma 1.32 mm long, 0.80 mm high; pronotum large, mesonotum smooth with notauli, mesoscutum nearly as long as metanotum in lateral view; propodeum longer than metanotum. Metasoma oblong with first metasomal segment much narrower at base, 1.79 mm long. Ovipositor extending outside the abdomen, about half length of metasoma. Legs slender except hind femur swollen, shorter than tibia. Forewing with pterostigma very narrow, 1-Rs longer than 1-M; 1cu-a antefurcal and shorter than 1-M; 1+2r longer than 3r, 2r-rs straight and long, more than 2× as long as 2r-m; 2-Rs+M 2.4× as long as 1-Rs+M, 2-Rs bent and longer than 3-Rs, 2r-m nearly as long as 3-Rs; 1m-cu shorter than 1-M, 2m-cu nebulous.

**Figure 4:**
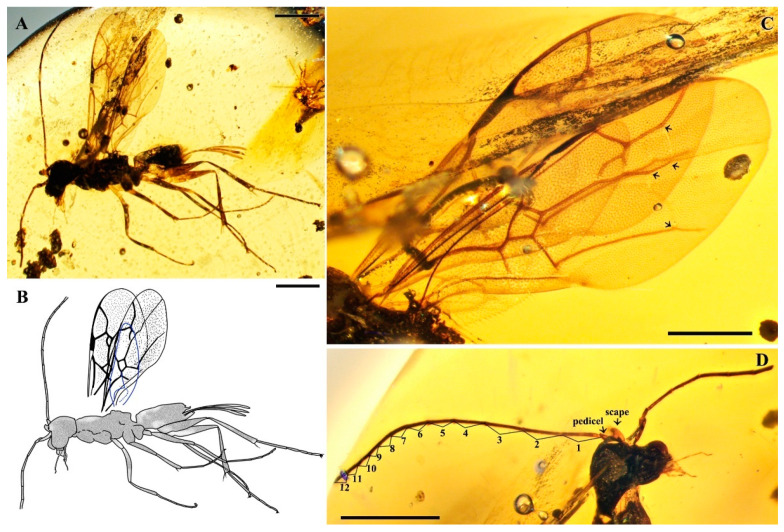
*Guyotemaimetsha ortegablancoi* Li, Kopylov and Rasnitsyn, sp. nov. Holotype GAU-HYM-MA-2016011. (**A**,**B**) Habitus, lateral views; scale bars = 1 mm. (**C**) Wings; (**D**) head; scale bars = 0.5 mm.

*Guyotemaimetsha ortegablancoi* Li, Kopylov and Rasnitsyn, sp. nov. (Figure 4).

urn:lsid:zoobank.org:act:334002DF-51CF-4161-96D3-8C0D4A40D405.

Type material. Holotype, Female, GAU-HYM-MA-2016011, well-preserved except for one antenna incomplete and wings incompletely visible because of irregularity of amber.

Etymology. The species is named in honor of the Spanish paleoentomologist Jaime Ortega-Blanco, who was the first to describe maimetshids from Spanish amber.

Locality and horizon. Hukawng Village, Kachin State, Northern Myanmar; mid-Cretaceous.

Diagnosis. Antennae with 14 segments as preserved. Forewing with 2-Rs and 3-Rs forming an obtuse angle, 2r-m nearly in line with 3-Rs, cell 3rm 1.5× as long as broad. Ovipositor longer than metasoma.

Description. Female. Body mainly dark, about 3.4 mm long (from antennae base to the end of metasoma); forewing length, 3.5 mm; width, 1.3 mm. Hind wing length, 2.0 mm. Head, 0.7 mm long and 1.0 mm high; antenna filiform, about 3.8 mm long, with 14 segments as preserved, scape slightly longer and wider than pedicel; five maxillary palpomeres. Both mesosoma and metasoma laterally slender, longer than high. Metasoma nearly rectangular except narrower at base, ovipositor extending outside the abdomen for more than metasoma length. Legs slender, hind legs with femur swollen except distally. Forewing with pterostigma narrow, 1-Rs nearly as long as 1-M; 1cu-a shorter than 1-M; 2r-rs long and straight, 2r-m in line with 3-Rs, nearly as long as 3-Rs, 2-Rs longer than 3-Rs; 2-Rs+M 2.8× as long as 1-Rs+M; 2m-cu absent.

Genus *Crucimaimetsha* Li, Kopylov and Rasnitsyn gen. nov.

Type species *Crucimaimetsha nigra* Li, Kopylov and Rasnitsyn, sp. nov.

Etymology. The new generic name is a combination of *crucis* (the Latin for cross), referring to the shape formed by 2rs-m, r-rs, 2-Rs, and 3-Rs and the generic name *Maimetsha*. Gender feminine.

Diagnosis. Forewing with 2-Rs+M slightly longer than 1-Rs+M, 1-M and 1m-cu not distinctly diverging, cell 2rm not petiolate, 3r-m absent.

Species included. Type species.

**Figure 5:**
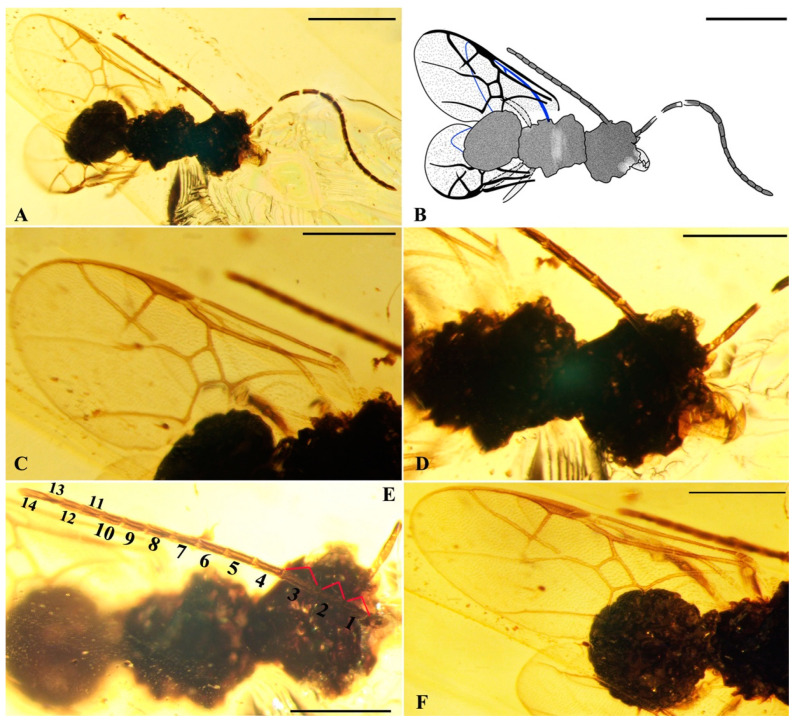
*Crucimaimetsha nigra* Li, Kopylov and Rasnitsyn, sp. nov. Holotype GAU-HYM-MA-2016012. (**A**,**B**) Habitus, lateral views. Scale bars = 1 mm. (**C**) Wings. (**D**) head. (**E**) Antennae. (**F**) Metasoma. Scale bars = 0.5 mm.

*Crucimaimetsha nigra* Li, Kopylov and Rasnitsyn, sp. nov. (Figure 5).

urn:lsid:zoobank.org:act:5D77419E-FBFC-4B1D-B8BB-DF97E98CA82C.

Type material. Holotype, sex unknown, GAU-HYM-MA-2016012, well-preserved except whole body very dark obscuring details of morphology and legs partly preserved.

Etymology. The specific name nigra is the Latin for black, referring to the body of wasp all black.

Locality and horizon. Hukawng Village, Kachin State, Northern Myanmar; mid-Cretaceous.

Diagnosis. As for the genus, by monotypy.

Description. Sex unknown. Body dark, wings hyaline. Body length about 2.3 mm (from the mandible to the end of metasoma); forewing length, 2.0 mm; forewing width, 0.9 mm. Head rounded, 0.6 mm long and 0.8 mm wide, mandible long extending, with three sharp teeth, apical longest, extending beyond contralateral one when closed; antenna filiform about 2.2 mm long, with 16 segments. Mesosoma narrower and longer than metasoma in dorsal view as preserved. Legs slender. Forewing with pterostigma very narrow, 1-Rs shorter than 1-M; 1cu-a shorter than 1-M; 2r-rs straight, slightly longer than full length of 2r-m (including its spectral part) and nearly in line with it; 2-Rs+M slightly longer than 1-Rs+M, 3-Rs dot-like short, 2m-cu absent. Hind wing with basal cell closed, very high, and apparently with vein A present.

Key to genera and species of Maimetshidae (modified after Engel, 2016) include the following:

1. Metasoma long and tubular, ovipositor sheath more than 3× as long as metatibia; metatibia distinctly clavate; forewing with r-rs joining pterostigma before its midlength, 1cu-a strongly postfurcal, 2m-cu entirely lost, Cu behind 2cu-a short, nebulous [Zorophratrinae; Barremian; Lebanon amber]………………………..*Zorophratra corynetes* Engel, 2016

—. Metasoma fusiform or globular, ovipositor sheath less than 2× as long as metatibia; metatibia not clavate; forewing with r-rs joining pterostigma behind its midlength, 1cu-a slightly antefurcal, 2m-cu usually present (in some species nebulous or entirely absent), Cu behind 2cu-a long, usually tubular [Maimetshinae; Barremian–Campanian]……………………………………………………………………………………………………………………………….2

2(1). Forewing with 2-Rs+M absent or at most 0.2× as long as 1-Rs+M, 1m-cu effectively confluent with 2Rs [*Turgonalus*; Barremian–Aptian]………………………………………………………………………………………………………………………3

—. Forewing with 2-Rs+M longer than 0.2× length of 1-Rs+M…………………………………………………………………..5

3(2). Forewing with cell 3r 1.2× as long as pterostigma, 2-Rs almost straight, as long as 4-Rs, M+Cu bowed next to 1cu-a; forewing length, 2.2 mm [Barremian; England]……………………………………...*Turgonalus cooperi* Rasnitsyn et al., 1998

—. Forewing with cell 3r at least 1.8× as long as pterostigma, 2-Rs bowed basally, 1.2× as long as 4-Rs, M+Cu nearly straight or evenly bowed; forewing length at least 3.6 mm………………………………………………………………………...4

4(3). Forewing with cell 2rm petiolate (r-rs joins Rs distad 2r-m), 3r-m oblique, 3-M 2× as long as 4-M [Barremian; Lebanon]……………………………………………………………………………………..*Turgonalus najlae* (Perrichot et al., 2011)

—. Forewing with cell 2rm sessile (r-rs aligned with 2r-m), 3r-m subvertical, 3-M as long as 4-M [Aptian; Russia]……………………………………………………………………………………………….*Turgonalus minor* Rasnitsyn, 1990

5(2). Forewing with single r-m (3r-m) meeting M behind r-rs and 2m-cu (2r-m lost) [*Maimetsha*; Santonian; Taimyr amber]……………………………………………………………………………………………….*Maimetsha arctica* Rasnitsyn, 1975

—. Forewing with either two nebulous or tubular r-m, or with single one (2r-m) meeting M before or opposite to r-rs and 2m-cu (3r-m lost)……………………………………………………………………………………………………………………6

6(5). 2r-m meeting Rs at or behind r-rs (cell 2rm sessile)…………………………………………………………………………7

—. 2r-m meeting Rs distinctly before r-rs (cell 2rm petiolate)…………………………………………………………………...9

7(6). Forewing with 3r-m absent, cell 1mcu as long as broad [*Crucimaimetsha*; Cenomanian; Kachin amber]………………………………………………………………….*Crucimaimetsha nigra* **Li, Kopylov and Rasnitsyn, sp. nov.**

—. Forewing with 3r-m present, cell 1mcu longer than broad…………………………………………………………………..8

8(7). 1-Rs slightly proclined [*Cretogonalys*; Cenomanian; Taimyr amber]…………*Cretogonalys taimyricus* Rasnitsyn, 1977

—. 1-Rs reclined [*Maimetshorapia*; Turonian; Botswana……………..*Maimetshorapia africana* Rasnitsyn and Brothers, 2009

9(6). Forewing with 2r-m shorter than petiole (3-Rs), cell 1mcu almost twice as long as high; 1-Cu longer than 2-Cu; pedicel comma-shaped in lateral view, [*Ahstemiam*; Campanian; Canada amber]…………………………………………………………………………………...*Ahstemiam cellula* McKellar and Engel, 2011

—. Forewing with 2r-m longer than petiole (3-Rs), cell 1mcu usually less long, 1-Cu rarely longer than 2-Cu, when 1mcu long (*Maimetshasia*), 1-Cu shorter than 2-Cu; pedicel fusiform…………………………………………………………….10

10(9). Forewing with 3r-m visible as nebulous or tubular vein………………………………………………………………...11

—. Forewing with 3r-m spectral or absent, at most visible as short stubs on, or just bends of, Rs and M at its place………………………………………………………………………………………………………………………………………17

11(10). Forewing with 4-Rs significantly longer than 3+4-M; costal cell distinctly broad, broader than pterostigma; 2rm right-triangular; 1-Rs+M 1.2× as long as 1-Cu [*Andyrossia*; Barremian; Weald Clay, England]……………………………………………………………………..*Andyrossia joyceae* Rasnitsyn and Jarzembowski, 1998

—. Forewing with 4-Rs as long as, or shorter than 3+4-M; costal cell narrow, about as wide as pterostigma; 2rm usually elongate-triangular; 1-Rs+M usually as long as 1-Cu………………………………………………………………………………12

12(11). Forewing 2Rs+M subequal to, or distinctly longer than, 1m-cu; cell 1rm shorter than 2rm [*Afrapia*]…………….13

—. Forewing 2Rs+M distinctly shorter than 1m-cu; cell 1rm longer than 2rm [*Iberomaimetsha*]…………………………...16

13(12). Notauli strongly diverging anteriorly [Turonian; Botswana]………*Afrapia robusta* (Rasnitsyn and Brothers, 2009)

—. Notauli more or less parallel……………………………………………………………………………………………………14

14(13). Forewing with 2r-m strongly reclined, 1-Rs+M parallel to r-rs, cell 2cua wider than 1mcu [Albian; Spain amber]…………………………………………………………………………………..*Afrapia nihtmara* (Ortega-Blanco et al., 2011)

—. Forewing with 2r-m subvertical, 1-Rs+M not parallel to r-rs, cell 2cua as wide as 1mcu……………………………….15

15(14). Forewing with pterostigma distinctly shorter than distance between base of Rs and 2r–rs, 4-Rs as long as 3+4-M [Turonian; Botswana]………………………………………………………………*Afrapia globularis* Rasnitsyn and Brothers, 2009

—. Forewing with pterostigma as long as distance between base of Rs and 2r–rs, 4-Rs shorter than 3+4-M [Turonian; Botswana]…………………………………………………………………………..*Afrapia variicornis* Rasnitsyn and Brothers, 2009

16(12). Forewing with cell 3rm as long as broad, 3-M as long as 4-M; mandibles symmetrical, both are 3-toothed [Albian; Spain amber]………………………………………………………...*Iberomaimetsha rasnitsyni* Ortega-Blanco et al., 2011

—. Forewing with cell 3rm shorter than broad, 3-M 2× as long as 4-M; mandibles asymmetrical, right 4-toothed, left 3-toothed [Santonian; Taimyr amber]……………………………………….*Iberomaimetsha pallida* Perrichot and Perkovsky, 2016

17(10). Forewing with 2-Rs+M shorter than 1-Rs+M, 1-M and 1m-cu distinctly diverging [*Maimetshasia*; Cenomanian; Kachin amber]…………………………………………………………………………………………………………………………..18

—. Forewing with 2-Rs+M longer than 1-Rs+M, 1-M and 1m-cu not distinctly diverging [*Guyotemaimetsha*; Albian-Cenomanian; French and Kachin amber]……………………………………………………………………………………………20

18(17). Antenna with 11 flagellomeres; forewing with 2r-m nearly as long as petiole (3-Rs); 2-Cu as long as 1m-cu…………………………………………………………………………..*Maimetshasia nova* **Li, Kopylov and Rasnitsyn, sp. nov.**

—. Antenna with 14 flagellomeres; forewing with 2r-m at least 2.5× as long as petiole (3-Rs); 2-Cu at least 1.8× as long as 1m-cu……………………………………………………………………………………………………….…………………………19

19(18). Forewing with 1-Rs+M 2× as long as 2-Rs+M, 2m-cu joins M behind 2r-m……………………………………………..

……………………………………………………………………………………………...*Maimetshasia kachinensis* Perrichot, 2013

—. Forewing with 1-Rs+M 1.3× as long as 2-Rs+M, 2m-cu joins M opposite 2r-m……………………………………………..

………………………………………………………………………….*Maimetshasia engeli* **Li, Kopylov and Rasnitsyn, sp. nov.**

20(17). Forewing with 4-Rs 2× as long as r-rs [Albian-Cenomanian; French amber]…………………………………………..

………………………………………………………………………………… ..*Guyotemaimetsha enigmatica* Perrichot et al., 2004

—. Forewing with 4-Rs as long as r-rs……………………………………………………………………………………………..21

21(20). Forewing with cell 3rm 1.5× as long as broad, 2-Rs+M 1.9× as long as 3-Cu [Cenomanian; Kachin amber]………………………………………………………..*Guyotemaimetsha ortegablancoi* **Li, Kopylov and Rasnitsyn, sp. nov.**

—. Forewing with cell 3rm as long as broad, 2-Rs+M 1.1–1.2× as long as 3-Cu………………………………………………22

22(21). Forewing with 3-M shorter than 4-M, 2-Rs+M 1.5× longer than cell 1mcu length [Cenomanian; Kachin amber]……………………………………………………………………………………..*Guyotemaimetsha concava* (Perrichot, 2013)

—. Forewing with 3-M longer than 4-M, 2-Rs+M 1.1× longer than cell 1mcu length [Cenomanian; Kachin amber]…………………………………………………………...*Guyotemaimetsha perrichoti* **Li, Kopylov and Rasnitsyn, sp. nov.**

## 4. Discussion

In our study, the taxonomy of Maimetshidae has been significantly revised. This was driven by two main reasons. First, the taxonomy of Maimetshidae was overly skewed towards genera. Of the 15 genera recognized before our revision, 13 were monotypic. At the same time, the differences between some genera were hardly convincing, even at the species level. Second, the taxonomy of the family disproportionately relied on body morphology characters, neglecting wing venation. While this approach is justified for extant insects, it is less applicable to the fossil record, where specimens with well-preserved bodies are not always available, and isolated wings often dominate. Body morphology characters preserved in amber are undoubtedly crucial for comparative anatomy and evolutionary biology. However, for practical purposes of identifying fossil insects, priority should undoubtedly be given to wing characters.

In this study, we synonymized several genera of Cretaceous Maimetshidae. Below, we justify our taxonomic decisions.

*Turgonalus* = *Turgonaliscus* syn. nov. = *Ahiromaimetsha syn. nov.*

These three genera (each with one species) comprise the tribe Maimetshini sensu Engel, 2016. Based on wing venation, this group is well-separated from other Maimetshidae. However, the differences within the group are limited to minor morphometric variations in wing venation. Two of the three species in this group are represented by compression fossils. *Turgonalus minor* is known from an impression of an entire insect with a poorly preserved body, while *Turgonalus* (=*Turgonaliscus*) *cooperi* is represented by an isolated wing. *Turgonalus* (*=Ahiromaimetsha*) *najlae* is an amber specimen with excellent preservation, but its body morphology characters cannot be compared with the other two species. When *Ahiromaimetsha* was described [7], *Turgonalus* and *Turgonaliscus* were still considered part of Trigonalidae [3,5] and were formally transferred to Maimetshidae only later [9]. Both *Turgonalus minor* and *Turgonalus* (=*Turgonaliscus*) *cooperi* fit the diagnosis of *Ahiromaimetsha* proposed by Perrichot et al., 2011. The separation of *Turgonalus cooperi* into a distinct genus, *Turgonaliscus* [9], appears insufficiently justified. Engel’s distinction is based on three wing venation characters: arched M+Cu, the presence of 3-Rs (petiole), and the absence of 2m-cu in *T. cooperi*. However, these characters do not work reliably within this group. M+Cu is also arched in *T. minor* (although this is less apparent in the figure by Rasnitsyn, 1990, it is visible in the holotype). The 2m-cu in *T. cooperi* is present, though nebulous, a feature common in many Maimetshidae, including all three members of Ahiromaimetshini. The presence of a short 3-Rs is unlikely to be a reliable generic character, as the appearance or disappearance of such short petioles often occurs even within species-level variation (e.g., in extant Ichneumonidae). Therefore, we conclude that these three species should be united under the genus *Turgonalus*. The differences among them are on the borderline of normal intraspecific variation. Retaining the tribe Ahiromaimetshini for a single genus seems unnecessary.

*Afrapia* = *Afromaimetsha* syn. nov. = *Iberomaimetsha* (part)

The genera *Afrapia* and *Afromaimetsha* were described from compression fossils from the Upper Cretaceous of Botswana [9]. These two genera share very similar wing venation, with the only significant difference being the shape of the notauli (converging in *Afromaimetsha* and parallel in *Afrapia*). While thoracic suture structure has been vital in the taxonomy of extant Hymenoptera, it is often impossible to assess in compression fossils. Even in the holotype of *A. variicornis* (preserved in profile), this character cannot be evaluated with confidence. The description of *Iberomaimetsha* from Spanish and Taimyr amber [4,7] introduced further uncertainty. The genus diagnosis aligns closely with *Afrapia* and *Afromaimetsha*, differing from *Afrapia* only in the presence of a prestigma (a thickening of R1 before the pterostigma) and from *Afromaimetsha* in the parallel notauli. However, *Afrapia* also has a prestigma ([8]: Figures 1a, 2a, 5a, and 6a), as do most other Maimetshidae. Our doubts about using notauli structure have been outlined above. Thus, synonymizing *Afrapia*, *Afromaimetsha*, and *Iberomaimetsha* seems justified. However, we observed a unique feature in the venation of *I. rasnitsyni* and *I. pallida*: these two species have a noticeably shortened 2-Rs+M, a rare trait among Maimetshidae. Based on this feature, we propose retaining *Iberomaimetsha* for these two species while transferring *I. nihtmara* to *Afrapia* (=*Afromaimetsha syn. nov.*). Additionally, the paratype of *I. nihtmara* ([7]: Figure 10) deserves attention. This specimen has incomplete wing preservation but shows venation distinct from the holotype (e.g., the shape of 2rm, arched M+Cu). This specimen may represent a different species of *Afrapia*, but its incomplete preservation precludes a formal description.

*Guyotemaimetsha* = *Burmaimetsha* syn. nov.

Perrichot [10] noted the similarity between these two genera. Differences included head structure (mandible size and antenna morphology) and wing venation details. While the two genera were initially distinguishable, new discoveries described here have filled the gap between them, rendering their separation unreliable. Consequently, we synonymize these two genera.

As a result of our revision and incorporating new discoveries, the family Maimetshidae now includes 2 subfamilies, 12 genera, and 23 species (Table 1). Seven genera remain monotypic, but they are now better diagnosed. The new system is based on wing venation characters, making it applicable to both amber and compression fossils.

Based on the review of Maimetshidae as shown in Table 1, we provided forewing line drawings of all 12 genera with 23 species, including five new species in this paper. Fossil Maimetshidae species varying in venation characters are as follows, which are considered to be diagnostic for interspecific differences of the forewings of Maimetshidae:

(1) two states for crossvein 1cu-a vs. 1-M: 1cu-a postfurcal only in *Zorophratra corynetes*, which is widely seen in Symphyta [23], but 1cu-a antefurcal in all other 22 species (e.g., *Afrapia globularis*);
(2) two states for crossvein 3r-m: 3r-m present in 5 genera with 11 species (e.g., *Turgonalus najlae*); 3r-m absent in 7 genera with 12 species (e.g., *Zorophratra corynetes*). More specifically, all 3 genera with 7 species from Myanmar lack 3r-m (e.g., *Crucimaimetsha nigra*);
(3) three states for 1-Rs+M and 2-Rs+M: 7 genera with 15 species have 1-Rs+M longer than 2-Rs+M (e.g., *Zorophratra corynetes*). Two genera with two species have 1-Rs+M nearly as long as 2-Rs+M (e.g., *Maimetshorapia africana*). Two genera with three species have 1-Rs+M shorter than 2-Rs+M (e.g., *Afrapia robusta*). Only one genus with three species (*Turgonalus najlae*, *T. cooperi* and *T. minor*) in Early Cretaceous without 2-Rs+M;
(4) three states for 2r-m: 2r-m nearly as long as 2r-rs (e.g., *Ahiromaimetsha cooperi*); 2r-m shorter than 2r-rs (e.g., *Zorophratra corynetes*); 2r-m very short, less than half length of 2r-rs (e.g., *Maimetshasia nova*);
(5) two states of cell 1mcu: 1mcu like big rectangle or trapezoidal (e.g., *Turgonalus najlae*); 1mcu like small square (e.g., *Crucimaimetsha nigra*).

The distribution of Maimetshidae across time and space is noteworthy (Table 1). The majority of records are concentrated around the middle of the Cretaceous (Albian through Turonian), with 14 species reported from Spanish, French, and Burmese ambers, as well as from Botswana shales. Interestingly, no records exist from the Cenomanian amber of Agapa or the Turonian amber of New Jersey. Earlier records, from Lebanese amber and the English and Siberian (Turga) shales of the Barremian, Berriasian, and Aptian ages, account for only five species in total. Similarly, four species are recorded from later periods, in the Santonian of Siberia and the Campanian of Canada. Notably, until the Campanian, all Maimetshidae records are from the Old World, which may suggest a late arrival of the family in the Americas.

Unfortunately, there is little we can infer about the biology of Maimetshidae beyond their parasitoid development, which can be deduced from their taxonomic position and the presence of a thin, flexible, external ovipositor. Additionally, it is reasonable to suppose that they were forest dwellers, as has been suggested previously for Evaniidae [24]. This hypothesis is supported mainly by the predominance of amber-preserved maimetshid fossils, although this prevalence is at least partially influenced by taphonomic factors (cf. Introduction).

## 5. Conclusions

Based on five well-preserved specimens of Maimetshidae from mid-Cretaceous Kachin amber, we have described a new genus along with five new species. The genera *Ahiromaimetsha* and *Turgonaliscus* are synonymized with *Turgonalus*, *Burmaimetsha* with *Guyotemaimetsha*, and *Afromaimetsha* with *Afrapia*. Additionally, *Iberomaimetsha nihtmara* has been transferred to *Afrapia*. This results in several new combinations:

*Turgonalus cooperi* (Rasnitsyn & Jarzembowski, 1998) comb. nov resurr;

*Turgonalus najlae* (Perrichot, Azar, Nel & Engel, 2011) comb. Nov;

*Afrapia robusta* (Rasnitsyn & Brothers, 2009) comb. Nov;

*Afrapia nihtmara* (Ortega-Blanco, Delclòs & Engel, 2011) comb. Nov.

We have included a table with forewing line drawings, locality and horizon information, and key wing venation characters for all fossil species to facilitate discussion of their differences. Additionally, we updated the key to genera originally proposed by Engel [9] and expanded it to species-level.

## Figures and Tables

**Table 1:** System and forewing venation of Maimetshidae (the illustrations are sourced from the respective papers).

Taxa and Forewing	Locality and Burial Medium	Horizon	Key Characters
Subfamily Zorophratrinae Engel, 2016			
1. *Zorophratra* Engel, 2016			
*Zorophratra corynetes* Engel, 2016 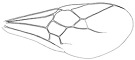	Lebanon, amber	Early Cretaceous (Barremian)	1cu-a postfurcal; 1Rs+M longer than 2Rs+M; 2r-m long; 3r-m absent.
Subfamily Maimetshinae Rasnitsyn, 1975			
2. *Turgonalus* Rasnitsyn, 1990			
*Turgonalus minor* Rasnitsyn, 1990 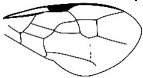	Russia, compression fossil	Early Cretaceous (Aptian)	1cu-a antefurcal; 1Rs+M long; 2Rs+M absent; 2r-m and 3r-m present and long.
*Turgonalus cooperi* Rasnitsyn and Jarzembowski, 1998, comb. resurr 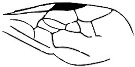	England, compression fossil	Early Cretaceous (Barremian)	1cu-a antefurcal; 1Rs+M long; 2Rs+M absent; 2r-m and 3r-m present and long.
*Turgonalus najlae* (Perrichot, Azar, Nel & Engel, 2011) comb. nov 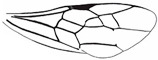	Lebanon, amber	Early Cretaceous (Barremian)	1cu-a antefurcal; 1Rs+M long; 2Rs+M absent; 1r-m and 2r-m present and long.
3. *Afrapia* Rasnitsyn and Brothers, 2009			
*Afrapia globularis* Rasnitsyn and Brothers, 2009 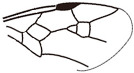	Bostwana, compression fossil	Late Cretaceous (Turonian)	1cu-a antefurcal; 1Rs+M nearly as long as 2Rs+M; 2r-m short, 3r-m long, 2m-cu absent.
*Afrapia variicornis* Rasnitsyn and Brothers, 2009 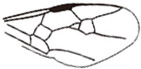	Bostwana, compression fossil	Late Cretaceous (Turonian)	1cu-a antefurcal; 1Rs+M shorter than 2Rs+M; 2r-m short, 3r-m long.
*Afrapia robusta* (Rasnitsyn & Brothers, 2009) comb. nov 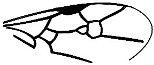	Bostwana, compression fossil	Late Cretaceous (Turonian)	1cu-a antefurcal; 1Rs+M shorter than 2Rs+M; 2r-m short, 3r-m long.
*Afrapia nihtmara* (Ortega-Blanco, Delclòs & Engel, 2011) comb. nov 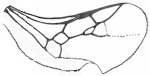	Spain, amber	Early Cretaceous (Albian)	1cu-a antefurcal; 1Rs+M shorter than 2Rs+M; 2r-m short, 3r-m long; 2m-cu absent.
4. *Ahstemiam* McKellar and Engel, 2011			
*Ahstemiam cellula* McKellar and Engel, 2011 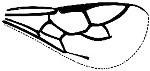	Canada, amber	Late Cretaceous (Campanian)	1cu-a antefurcal; 1Rs+M longer than 2Rs+M; 2r-m long, 3r-m absent.
5. *Andyrossia* Rasnitsyn and Jarzembowski, 1998			
*Andyrossia joyceae* Rasnitsyn and Jarzembowski, 1998 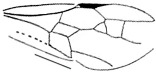	England, compression fossil	Early Cretaceous (Barremian)	1cu-a antefurcal; 1Rs+M longer than 2Rs+M; 2r-m short, 3r-m long.
6. *Guyotemaimetsha* Perrichot, Nel, and Néraudeau, 2004			
*Guyotemaimetsha enigmatica* Perrichot, Nel, and Néraudeau, 2004 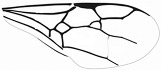	France, amber	Middle Cretaceous (Albian-Cenomanian)	1cu-a antefurcal; 1Rs+M shorter than 2Rs+M; 2r-m short, 3r-m absent; 2m-cu nebulous.
*Guyotemaimetsha concava* (Perrichot, 2013), comb. nov 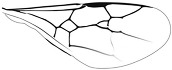	Myanmar, amber	Late Cretaceous (Cenomanian)	1cu-a antefurcal; 1Rs+M shorter than 2Rs+M; 2r-m short, 3r-m absent; 2m-cu nebulous.
*Guyotemaimetsha perrichoti* Li, Kopylov and Rasnitsyn, sp. nov. 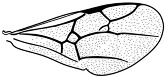	Myanmar, amber	Late Cretaceous (Cenomanian)	1cu-a antefurcal; 1Rs+M shorter than 2Rs+M; 2r-m short, 3r-m absent; 2m-cu nebulous.
*Guiyotemaimetsha ortegablancoi* Li, Kopylov and Rasnitsyn, sp. nov. 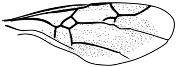	Myanmar, amber	Late Cretaceous (Cenomanian)	1cu-a antefurcal; 1Rs+M shorter than 2Rs+M; 2r-m short; 3r-m and 2m-cu absent with short traces points on two sides veins.
7. *Cretogonalys* Rasnitsyn, 1977			
*Cretogonalys taimyricus* Rasnitsyn, 1977 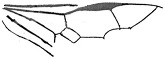	Taimyr, amber	Late Cretaceous (Santonian)	1cu-a antefurcal; 1Rs+M shorter than 2Rs+M.
8. *Iberomaimetsha* Ortega-Blanco, Perrichot, and Engel, 2011			
*Iberomaimetsha rasnitsyni* Ortega-Blanco, Perrichot, and Engel, 2011 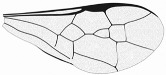	Spain, amber	Early Cretaceous (Albian)	1cu-a antefurcal; 1Rs+M longer than 2Rs+M; 2r-m short, 3r-m long; 2m-cu present.
*Iberomaimetsha pallida* Perrichot and Perkovsky, 2016 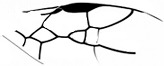	Taimyr, amber	Late Cretaceous (Santonian)	1cu-a antefurcal; 1Rs+M longer than 2Rs+M; 2r-m short, 3r-m long; 2m-cu present.
9. *Maimetsha* Rasnitsyn, 1975			
*Maimetsha arctica* Rasnitsyn, 1975 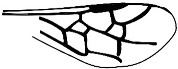	Taimyr, amber	Late Cretaceous (Santonian)	1cu-a antefurcal; 1Rs+M shorter than 2Rs+M; 2r-m short, 3r-m absent; 2m-cu present.
10. *Maimetshasia* Perrichot, 2013			
*Maimetshasia kachinensis* Perrichot, 2013 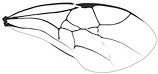	Myanmar, amber	Late Cretaceous (Cenomanian)	1cu-a antefurcal; 1Rs+M longer than 2Rs+M; 2r-m short, 3r-m absent; 2m-cu nebulous.
*Maimetshasia nova* Li, Kopylov and Rasnitsyn, sp. nov. 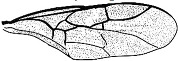	Myanmar, amber	Late Cretaceous (Cenomanian)	1cu-a antefurcal; 1Rs+M longer than 2Rs+M; 2r-m short, 3r-m absent; 2m-cu present.
*Maimetshasia engeli* Li, Kopylov and Rasnitsyn, sp. nov. 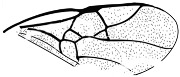	Myanmar, amber	Late Cretaceous (Cenomanian)	1cu-a antefurcal; 1Rs+M longer than 2Rs+M; 2r- m short, 3r-m absent; 2m-cu nebulous.
11. *Maimetshorapia* Rasnitsyn and Brothers, 2009			
*Maimetshorapia africana* Rasnitsyn and Brothers, 2009 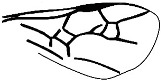	Bostwana, compression fossil	Late Cretaceous (Turonian)	1cu-a antefurcal; 1Rs+M nearly as long as 2Rs+M; 2r-m and 3r-m long.
12. *Crucimainestsha* gen. nov.			
*Crucimainestsha nigra* Li, Kopylov and Rasnitsyn, sp. nov. 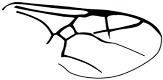	Myanmar, amber	Late Cretaceous (Cenomanian)	1cu-a antefurcal; 1Rs+M nearly as long as 2Rs+M; 2r-m short, nearly in line with 2r-rs; 3r-m absent, 2m-cu absent.

## Data Availability

All data from this study are available in this paper and the associated papers.
